# Oncogenic comparison of human papillomavirus type 58 E7 variants

**DOI:** 10.1111/jcmm.14059

**Published:** 2018-12-21

**Authors:** Priscilla TY Law, Siaw Shi Boon, Chenghua Hu, Raymond WM Lung, Grace PY Cheung, Wendy CS Ho, Zigui Chen, Paola Massimi, Miranda Thomas, David Pim, Lawrence Banks, Paul KS Chan

**Affiliations:** ^1^ Department of Microbiology The Chinese University of Hong Kong, Prince of Wales Hospital Shatin, New Territories, Hong Kong SAR China; ^2^ Department of Anatomical and Cellular Pathology The Chinese University of Hong Kong, Prince of Wales Hospital Shatin, New Territories, Hong Kong SAR China; ^3^ International Centre for Genetic Engineering and Biotechnology Trieste Italy

**Keywords:** HPV58, human papillomavirus, oncogenicity, T20I/G63S, variant

## Abstract

Human papillomavirus 58 (HPV58) ranks the second or third in East Asian cervical cancers. Current studies on HPV58 are scarce and focus on the prototype. Previously, we identified the three most common circulating HPV58 E7 strains contained amino acid alterations: G41R/G63D (51%), T20I/G63S (22%) and T74A/D76E (14%) respectively. Among them, the T20I/G63S variant (V1) had a stronger epidemiological association with cervical cancer. We therefore suggested that V1 possessed stronger oncogenicity than the other two variants. Here, we performed phenotypic assays to characterize and compare their oncogenicities with HPV58 E7 prototype. Our results showed that overexpression of V1 conferred a higher colony‐forming ability to primary murine epithelial cells than prototype (*P *<* *0.05) and other variants, implicating its higher immortalising potential. Further experiments showed that both V1 and prototype enhanced the anchorage‐independent growth of NIH/3T3 cells (*P *<* *0.001), implicating their stronger transforming power than the two other variants. Moreover, they possessed an increased ability to degrade pRb (*P *<* *0.001), which is a major effector pathway of E7‐driven oncogenesis. Our work represents the first study to compare the oncogenicities of HPV58 E7 prototype and variants. These findings deepened our understanding of HPV58 and might inform clinical screening and follow‐up strategy.

## 1 INTRODUCTION

Persistent infection with high‐risk human papillomavirus (HPV) is a necessary aetiological cause of cervical cancer, which is the fourth most common cancer in women worldwide.^1^ Like in other parts of the world, HPV16 and 18 are the two most prevalent HPV types and cause about 70% of invasive cervical cancers (ICC) in Asia. Among the other 10 HPV types that carry a clear carcinogenic potential (HPV31, 33, 35, 39, 45, 51, 52, 56, 58 and 59),^2^, ^3^, ^4^ HPV58 plays a special role in East Asia.^5^, ^6^, ^7^, ^8^, ^9^

The importance of HPV58 has been investigated by many research groups, and it has been unanimously concluded that HPV58 accounts for a remarkably higher proportion of cervical cancer in East Asia than elsewhere. For instance, HPV58 was found in 29% of HPV‐positive cervical squamous cell carcinomas (SCC) in Shanghai (East China),^10^ 21% in Taiwan,^11^ 19% in South Korea,^12^ 14% in Japan,^13^ 13% in south Sichuan (southwestern China),^14^ 10% in Hong Kong (east China)^15^ and 9% in Yangtze River Delta region (east China)^16^ but only 0.6%‐2.8% outside East Asia.^17^, ^18^ HPV58 is therefore the second or third most prevalent HPV type in East Asia,^3^, ^8^, ^18^ even though it only ranks sixth or seventh worldwide.^5^ Furthermore, our meta‐analysis scrutinising 219 publications from 1994 to 2012 found that attribution of HPV58 to cervical cancer was 3.7‐fold higher in East Asia than elsewhere, even after adjustment for bystander effect in multiple‐type infections.^19^

The reason for such ethnogeographical specificity in the disease burden associated with HPV58 remains unknown. However, unlike HPV16 and 18 in which the oncogenicity is well‐established^20^, ^21^, studies on HPV58 biology are scarce.^22^, ^23^ Current studies only show that HPV58 is less oncogenic than HPV16,^22^, ^23^, ^24^ but none of them focuses on investigating the oncogenicity of its variants. To remedy this lack, we carried out an epidemiological study on 1924 Hong Kong women and identified an HPV58 variant (E7 T20I/G63S, designated as V1) conferring an odds ratio of 26.8 for cervical intraepithelial neoplasia (CIN) III or SCC.^25^ This epidemiological association was subsequently reproduced by an independent research group from Zhejiang province, East China.^26^

We then expanded our epidemiological study to an international scale, using 401 HPV58‐containing clinical samples collected from 15 locations across four continents.^24^ We found that the three most common circulating HPV58 E7 strains contained the amino acid alterations: G41R/G63D (51%), T20I/G63S (V1; 22%) and T74A/D76E (14%) respectively.^27^ HPV58 E7 prototype (GenBank accession no. D90400), which was previously isolated from a Japanese woman suffering from invasive cervical carcinoma,^28^ was the fourth common strain within the population. Concordant with our previous finding, the HPV58 E7 V1 (T20I/G63S) variant conferred a significantly higher risk for CIN III and ICC (odds ratio: 4.44, *P *=* *0.007) compared to other HPV58 E7 variants.^27^

On the other hand, although E6 is another key HPV oncogenic protein, previous studies from us and others^27^ have concordantly shown that HPV58 E6 is relatively conserved compared to E7, and did not show any significant risk association. With this background, we suggested that the E7 T20I/G63S (V1) variant of HPV58 might possess a stronger oncogenicity than the other HPV58 E7 variants.

Therefore, this study aimed to investigate the comparative oncogenic properties of HPV58 E7 prototype and its variants, particularly the E7 T20I/G63S (V1), using a series of phenotypic and molecular assays. Our findings can help to translate our previous epidemiological observations into clinical use for improving HPV surveillance and therapeutic design, especially in East Asia.

## 2 MATERIALS AND METHODS

### 2.1 Cell lines

HEK293, HeLa, NIH/3T3 and U‐2 OS cells were purchased from the American Type Culture Collection (Manassas, VA, USA) and maintained in Dulbecco's modified eagle medium, supplemented with 10% FBS in a 37°C humidified incubator containing 5% CO_2_. Established human cell lines used in this study were short tandem repeat (STR) profiled using AmpFlSTR Identifiler^®^ Plus PCR Amplification Kit (ThermoFisher Scientific, Waltham, MA, USA) with the Applied Biosystems 3500 Series Genetic Analyzer and analysed by GeneMapper^®^ Software 5 (Applied Biosystems, Foster City, CA, USA). The STR profile of all cells lines showed >88% concordance with their reference profile in the ATCC cell line database.

### 2.2 Plasmids

HPV58 E7 prototype and variants were amplified from clinical specimens and cloned into the pcDNA3.1 mammalian expression vector (ThermoFisher Scientific) using primers 5′‐CGCGAATTCATGAGAGGAAACAACCCAAC‐3′ (sense) and 5′‐ATAAGAATGCGGCCGCTTATTGCTGTGCACAGCT‐3′ (antisense). The pcDNA3.1 vector contains a neomycin resistance gene, which allows positive clone selection by geneticin (G418) in subsequent experiments. Apart from E7 T20I/G63S (V1; worldwide the second most prevalent variant which contributed to 22% of cases), we also included E7 G41R/G63D (V2) and E7 T74A/D76E (V3), which were respectively the most prevalent variant (51%) and the third most common variant (14%) worldwide in our study.^27^ We also generated artificial single mutants, V1A (T20I) and V1B (G63S) in order to delineate their individual functional roles.

### 2.3 Sequence alignment and homology modelling

Sequence alignment of HPV58 E7 with HPV45 E7 and HPV16 E7 was performed by Clustal Omega multiple sequence alignment. The three‐dimensional (3D) structure of HPV58 E7 prototype was estimated by homology modelling with PyMOL^™^ using the published 3D structure of HPV45 E7 determined by X‐ray crystallography as reference.^29^

### 2.4 Immortalisation assay

The immortalising ability of HPV58 E7 was assayed using primary murine epithelial cells extracted from baby rat kidney (BRK) with a method modified from Massimi and Banks.^30^ In brief, HPV58 E7 constructs were transfected into BRK cells collected from 9‐day‐old Wistar Hannover rats by calcium precipitation. Activated H‐ras (EJ‐ras) was co‐transfected into each group to augment the transforming process.^31^ Successfully transfected cells were then selected by medium supplemented with 220 μg/mL G418 (ThermoFisher Scientific). As cultured normal cells have a finite lifespan, BRK cells transfected with only EJ‐ras eventually died within 7 days after selection. Cells were allowed to grow for 14 days further. Colonies consisting of at least 30 cells were counted and categorized into three groups: small (<6 mm^2^), medium (6‐21 mm^2^) and large (>21 mm^2^). A score named as colony formation index (CFI) was calculated for each variant based on colony number and size using the formula: no. of small colonies × 1 + no. of medium colonies × 4 + no. of large colonies × 8.

### 2.5 Colony formation assay in soft agar

HPV58 E7 constructs and LacZ were cotransfected into NIH/3T3 using Lipofectamine™ LTX transfection reagent (ThermoFisher Scientific). Successfully transfected cells were selected by medium supplemented with 500 μg/mL G418 (ThermoFisher Scientific) for 7‐10 days until colonies were over 50% confluent. One thousand transfected cells were then mixed with 0.35% low melting point agarose (ThermoFisher Scientific) and overlaid onto a 0.6% agarose base layer in a six‐well plate. Cells were allowed to grow for 2 weeks further and colonies consisting of at least 50 cells were counted under light microscopy. Colonies were finally stained with 0.1% *p*‐iodonitrotetrazolium violet (Sigma‐Aldrich, St. Louis, MO, USA) and images were captured by digital camera.

### 2.6 Immunofluorescence analysis

U2‐OS cells plated onto coverslips were transfected with HPV58 E7. Cells were fixed with cold absolute methanol 24 hours after transfection and blocked with 3% bovine serum albumin. Cells were then probed with specified primary antibody against HPV58 E7 (ThermoFisher Scientific), followed by Alexa Fluor^®^ 568‐conjugated anti‐rabbit secondary antibody (ThermoFisher Scientific) and counterstained in 4′,6‐diamidino‐2‐phenylindole (DAPI). The subcellular location of HPV58 E7 was examined under a confocal microscope (Leica, Wetzlar, Germany).

### 2.7 Protein stability analysis

The half‐life of different HPV58 E7 variants was examined by transfection of HPV58 E7 into HEK‐293 cells. Cycloheximide (CHX) (Sigma‐Aldrich), at a final concentration of 10 μg/mL was supplemented to halt protein translation 24 hours after transfection. Cell lysates were then harvested at 0, 30 minutes, 1, 2 and 3 hours following CHX treatment. Differences between HPV58 E7 prototype and variants on E7 stability were then compared using Western blot. E7 half‐life was then calculated with the one phase exponential decay function using GraphPad Prism 7.

### 2.8 Retinoblastoma protein (pRb) and pocket protein degradation assay

The effects of different variants on pRb and pocket protein degradation were examined by cotransfection of HPV58 E7, HA‐tagged pRb or pocket protein construct and LacZ into HEK‐293 cells. Whole cell lysates were harvested 24 hours after transfection and subjected to Western blot.

### 2.9 Western blotting

Twenty micrograms of total protein was resolved by sodium dodecyl sulphate‐polyacrylamide gel electrophoresis, and transferred onto a polyvinylidene fluoride membrane (GE Healthcare, Chicago, IL, USA). The membrane was then probed with specified primary antibodies overnight as follows: HPV16 E7 (Cervimax, Vienna, Austria), β‐galactosidase (Promega, Madison, WI, USA), HA (12CA5; Roche Diagnostics, Risch‐Rotkreuz, Switzerland), glyceraldehyde‐3‐phosphate (GAPDH) (Chemicon, Billerica, MA, USA) and β‐actin (C4, Santa Cruz Biotechnology, Dallas, TX, USA). HPV58 E7 polyclonal antibody was custom synthesized from immunising rabbits with HPV58 E7 peptides (aa3‐20) using a 90‐day protocol with three boosters (ThermoFisher Scientific). Blots were visualized using ECL chemiluminescence (GE Healthcare) and images were captured using ChemiDoc™ Imagine System (Bio‐Rad, Hercules, CA, USA). Actin or GAPDH served as loading control for the blot. Band Intensities were quantitated by ImageLab and normalized with corresponding loading control level.

### 2.10 Statistical analysis

All experiments were conducted in triplicate wells/dishes for at least three independent times. Data were expressed as mean ± SEM with statistical analyses being performed with GraphPad Prism 7. Effects of HPV58 E7 variations were compared against prototype and each other by one‐way ANOVA. A *P*‐value of <0.05 was considered to be statistically significant.

## 3 RESULTS

### 3.1 Location of the amino acid residues T20 and G63 within HPV58 E7 implies functional importance

**Figure 1:**
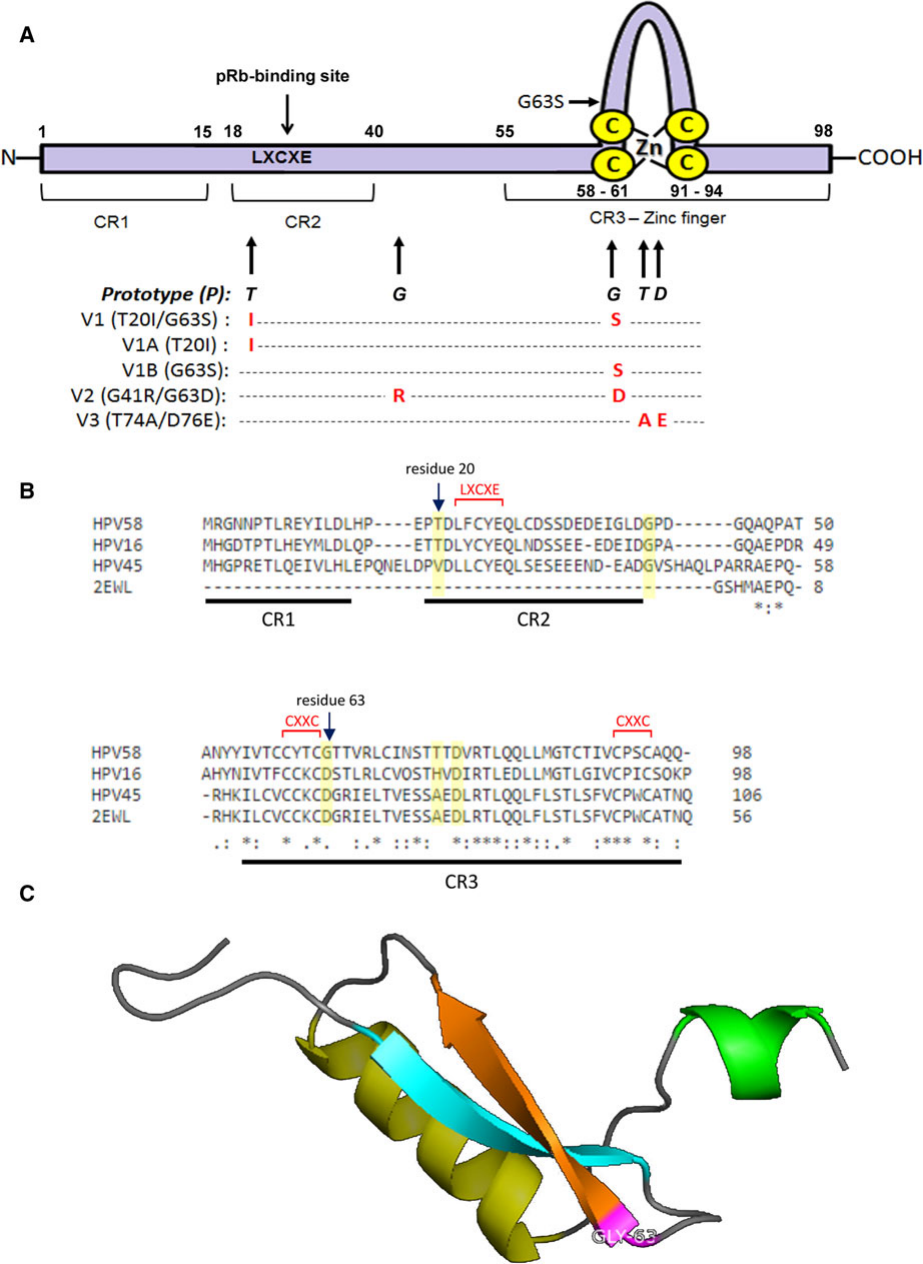
Amino acid sequence of HPV58 E7 prototype and its variants. (A) Schematic diagram shows the basic structure of E7 which contains three conserved regions (CR): CR1 and 2 core domain, and a C‐terminal CR3 or zinc finger domain. The LXCXE motif embedded within the CR2 domain is the region important for pRB and related pocket protein targeting. Our previous studies showed that the three most common circulating HPV58 E7 strains carry amino acid substitutions (in red font) from HPV58 E7 prototype (in black, bold italic font) as follows: threonine (T) at position 20 to isoleucine (I) and glycine (G) at position 63 to serine (S), or designated as T20I/G63S within *Variant 1* (V1); G at position 41 to arginine (R) and G at position 63 to aspartic acid (D), or designated as G41R/G63D within *Variant 2* (V2); T at position 74 to alanine (A) and D at position 76 to glutamic acid (E), or designated as T74A/D76E within *Variant 3* (V3). In this study, we also included artificial single mutants, *Variant 1A* (V1A; T20I) and *Variant 1B* (V1B; G63S) to delineate their individual functional roles. (B) Sequence alignment of HPV58 E7 with HPV16E7 and HPV45 E7 (reference model for homology modelling). Residue 20 of HPV58 E7 locates within CR 2 of E7, in close proximity to the conserved LXCXE motif known for retinoblastoma protein (pRB) targeting, whilst residue 63 resides within the zinc finger domain of CR3. (C) Three‐dimensional (3D) structure of HPV58 E7 was constructed by homology modelling using the published 3D structure of HPV45 E7 as reference. The predicted structure shows that G63 lies at the N‐terminal of one of the beta sheets on CR3, suggesting that a change in this amino acid residue might confer functional importance

Our previous epidemiological data collected from 401 HPV58‐containing clinical samples worldwide revealed that the three most common circulating E7 strains were variants containing the amino acid alterations: G41R/G63D (V2; 51%), T20I/G63S (V1; 22%) and T74A/D76E (V3; 14%)^27^ (Figure 1A). Among them, E7 T20I/G63S (V1) is of our particular interest, due to its stronger association with cervical cancers compared to other variants.^27^ In view of these, we first wanted to predict whether the two amino acid substitutions T20I and G63S in the V1 variant confer any possible functional importance based on their location within the HPV58 E7 protein.

E7 contains three conserved regions (CR), with CR1 and CR2 at the amino terminal, followed by CR3. Sequence alignment of HPV58 E7 to HPV16 E7 indicates that residue 20 of HPV58 E7 locates within CR2 of E7, in close proximity to the conserved LXCXE motif known for pRB targeting, whilst residue 63 resides within the zinc finger domain of CR3 (Figure 1A,B). On the other hand, G41R/G63D and T74A/D76E are located downstream of CR2, near or within CR3 of E7, which is further away from the important LXCXE motif (Figure 1A,B).

As the HPV58 E7 structure has not been established, we utilized in silico algorithms to predict its 3D structure by homology modelling. The 3D structures of HPV1 E7^32^ and HPV45 E7^29^ have been reported. HPV45 E7 (Protein Data Bank ID: 2EWL) was used as the reference model as it shares a higher sequence homology with HPV58 E7 when compared with HPV1 E7 (43% vs. 35%). X‐ray crystallography of the entire HPV45 E7 protein showed that its N‐terminal domain is unfolded. We therefore used the C‐terminal zinc‐binding domain of E7 for homology modelling in our studies (Figure 1C). The predicted 3D structure of HPV58 E7 revealed that G63 lies at one of the beta sheets within CR3, which implies that substitution of this amino acid residue might change the secondary protein structure and confer certain functionally important differences (Figure 1C). We then sought to determine the oncogenic potential of HPV58 E7 V1 (T20I/G63S) experimentally through phenotypic assays. As the E7 protein of high‐risk HPV is well known to be responsible for cellular transformation, the possible effects of HPV58 E7 variations on immortalisation and transforming ability were examined.

### 3.2 HPV58 E7 T20I/G63S variant increased primary murine epithelial cells immortalisation

**Figure 2:**
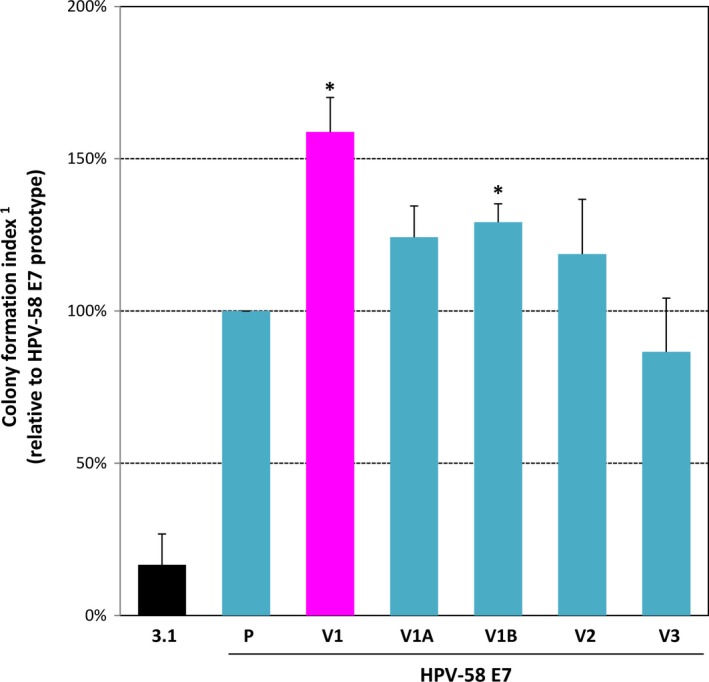
Increased colony‐forming ability of HPV58 E7 T20I/G63S variant in primary baby rat kidney (BRK) cells. Primary epithelial cells were obtained from 9‐day‐old Wistar Hannover rats and transfected with the HPV58 E7 expression plasmids, plus EJ‐ras and placed under G418 selection. After 14 days, colonies of at least 30 cells were counted and categorized into small (<6 mm^2^), medium (6‐24 mm^2^) and large (>24 mm^2^) size. A colony formation index (CFI) was calculated for each group by the equation: no. of small colonies × 1 + no. of medium colonies × 4 + no. of large colonies × 8. The experiment was conducted in triplicate dishes for at least three times. Data are expressed as average ± SEM from three independent experiments. Our results showed that HPV58 E7 (including prototype and all tested variants) could significantly induce colony formation in primary murine epithelial cells compared with cells transfected with EJ‐ras alone. The HPV58 E7 V1 (T20I/G63S) variant conferred a higher colony‐forming ability to the primary BRK cells as compared with prototype (P) (*P <* *0.05), or with other commonly circulating variants (V2: G41R/G63D; V3: T74A/D76E), implicating its higher immortalising power

We first compared the immortalising ability of different HPV58 E7 variants using primary BRK cells. In this assay, we measured the colony formation ability upon E7 and oncogenic H‐ras (EJ‐ras) expression using CFI. As cultured normal primary cells have a finite lifespan, BRK cells transfected with only EJ‐ras died within 7 days after selection. We observed that HPV58 E7‐transfected cell colonies started to appear 7‐10 days after selection. In line with our previous epidemiological observations, the HPV58 E7 V1 (T20I/G63S) variant displayed a higher colony‐forming ability by 45 ± 25% on day 14 of the immortalisation assay when compared with prototype (*P* < 0.05) and other variants in BRK cells suggesting that it has a higher immortalising and transforming potential (Figure 2).

### 3.3 HPV58 E7 T20I/G63S variant increased anchorage‐independent growth in soft agar

**Figure 3:**
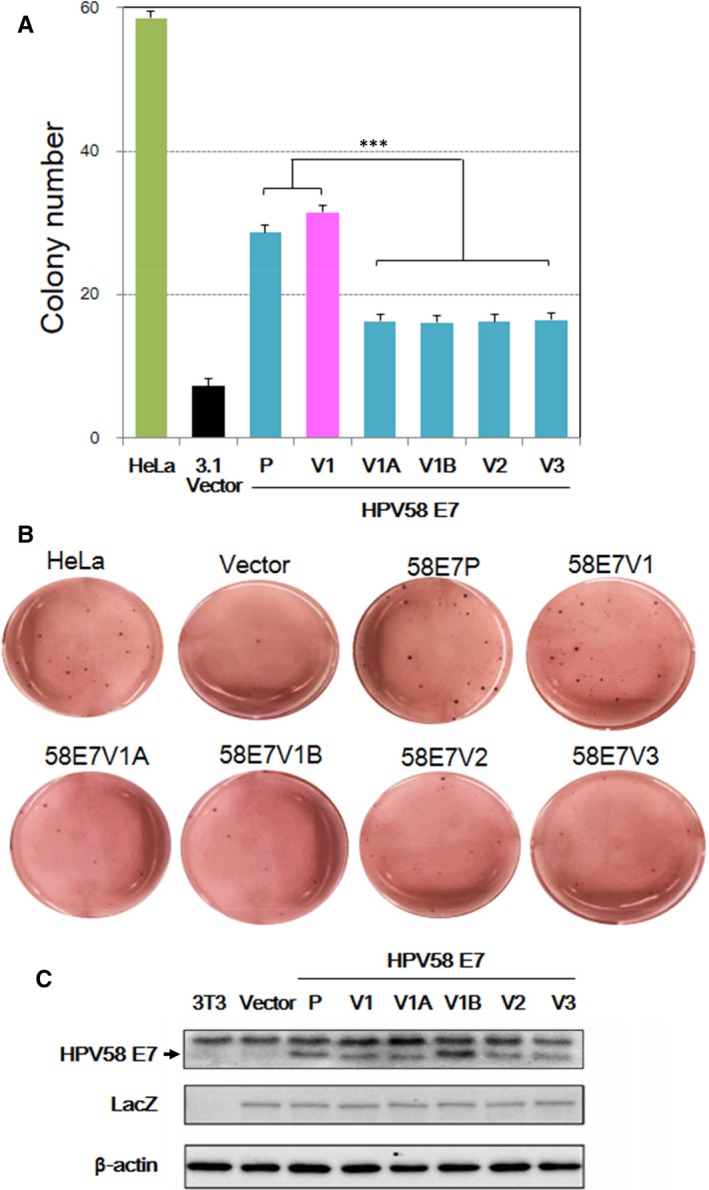
Higher colony‐forming ability of HPV58 E7 T20I/G63S variant and prototype in soft agar. The transforming ability of HPV58 E7 prototype and variants were assessed by comparing their anchorage‐independent growth in a soft agar colony formation assay. HPV58 E7 and LacZ were cotransfected into NIH/3T3 cells, selected under G418 for 1 week, and seeded into soft agar. After 2‐3 weeks, colonies of at least 50 cells were counted. (A) Results showed that HPV58 E7 (including prototype and all tested variants) could significantly induce colony formation in NIH/3T3 compared with cells transfected with LacZ alone. HPV58 E7 V1 (T20I/G63S) and prototype (P) demonstrated a significantly higher colony‐forming ability in soft agar than the other common circulating variants (V2: G41R/G63D; V3: T74A/D76E), indicating their higher transforming ability (***, *P* < 0.0001). Single amino acid variation of either T20I (V1A) or G63S (V1B) alone was not sufficient to induce a higher transforming ability than V2 or V3; while their combined effects allowed V1 to achieve a statistically stronger effect than individual variants. The experiment was conducted in triplicate wells for three times. Data are expressed as average ± SEM from three independent experiments. (B) Representative images of corresponding group are shown. (C) Western blotting confirmed expression of HPV58 E7, and LacZ showed equal transfection efficiency in different groups after G418 selection prior to plating into soft agar

Earlier studies clearly showed that HPV16 E7 can induce anchorage‐independent growth of murine fibroblast cells.^27^, ^28^ However, to date, transforming ability of HPV58 E7 has not been demonstrated. Therefore, we next wanted to study the ability of HPV58 E7 variants in transforming fibroblasts using soft agar colony formation assay, which is a stringent and commonly used in vitro transformation assay. In this assay, we included HeLa, an established HPV18‐containing cervical cancer cell line, as a positive control. The primary mouse embryonic fibroblast NIH/3T3 cells was chosen as the cell model as it can grow in monolayer, and can attain multilayer growth or form foci upon overexpression of oncogenes with transforming ability. Equal amount of HPV58 E7 variant constructs was transfected into NIH/3T3 cells. Our results showed that, similar to HeLa cells, HPV58 E7 colonies appeared 1 week after plating into the soft agar. On day 14, HPV58 E7 V1 (T20I/G63S) variant and prototype demonstrated a significantly higher colony number by 93 ± 2% and 76 ± 2%, respectively, compared with other variants (*P* < 0.0001) (Figure 3A,B). Whilst the two artificial mutants, E7 V1A (T20I) and V1B (G63S) showed similar number of colonies, and at similar levels as V2 (G41R/G63D) and V3 (T74A/D76E). Western blot confirmed the expression of HPV58 E7. Co‐expression of LacZ showed equal transfection efficiency among all tested groups and confirmed that the observed effects were bona fide and were not caused by different transfection efficiencies between variants (Figure 3C). Our results demonstrated that the HPV58 E7 V1 (T20I/G63S) variant attained a stronger ability to induce anchorage‐independent growth and possessed a higher transforming ability, similar to prototype when compared to other E7 variants. It seems that single amino acid variation of either T20I or G63S may not be sufficient to induce full transformation, while amino acid alteration at both sites allows HPV58 E7 to achieve higher transforming ability compared to other HPV58 E7 variants.

### 3.4 The effects of amino acid variation on biochemical properties of HPV58 E7

As we observed HPV58 E7 variants possess different immortalising and transforming ability, we proceeded to characterize the potential effects of these amino acid alterations on the biochemical properties of HPV58 E7, in the aspect of cellular localisation and protein turnover.

#### 3.4.1 HPV58 E7 prototype and variants localized predominantly in the nucleus with a punctate pattern

**Figure 4:**
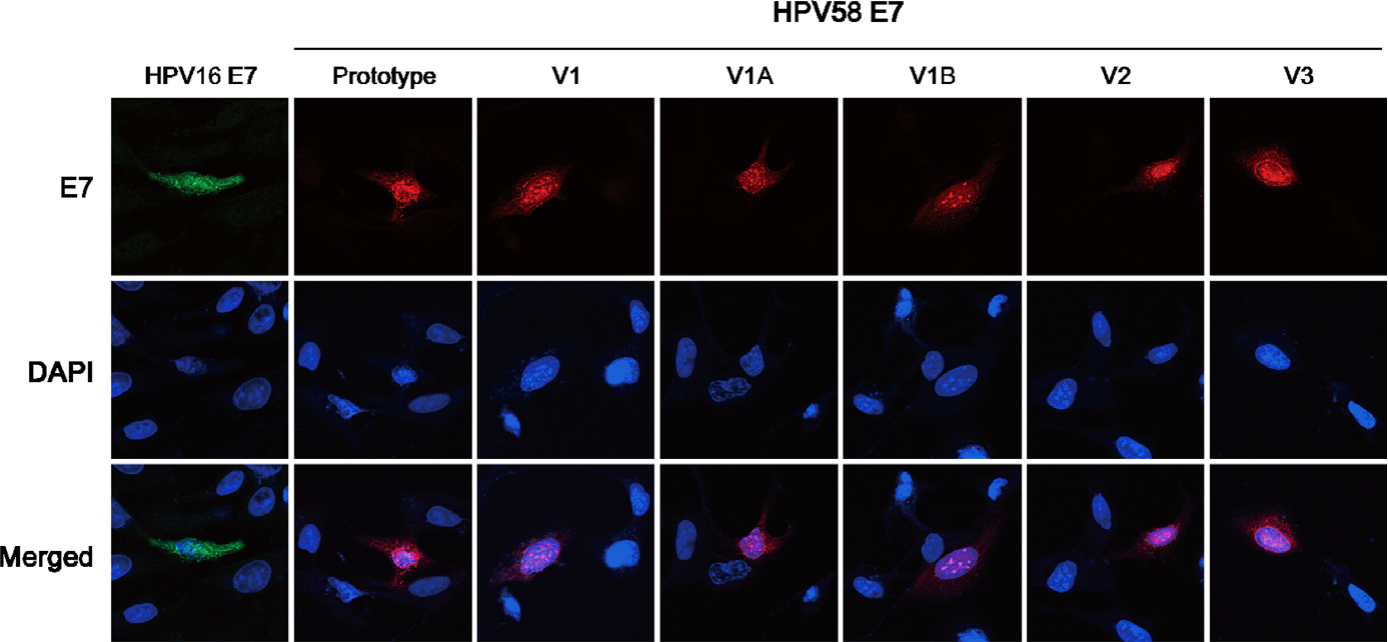
Subcellular localisation of HPV58 E7. The subcellular localisation of HPV58 E7 were examined by immunofluorescence using HPV58 E7 antibody, and AlexaFluor^®^‐568 conjugated secondary antibody 24 h after transfection in U‐2 OS cells. Confocal microscopy showed HPV58 E7 predominantly located in the nucleus with a punctate pattern and also found in the cytoplasm. In contrast, HPV16 E7 was distributed evenly throughout the cells in both nucleus and cytoplasm. Further subcellular localisation studies revealed that all the three other HPV58 E7 variants (V1: T20I/G63S; V2: G41R/G63D; V3: T74A/D76E) and the two artificial mutants (V1A: T20I only; V1B: G63S only) had subcellular localisation similar with that of prototype (P), implicating they might not affect the nuclear functions of HPV58

The subcellular localisation of a protein reflects its cellular targets and possible molecular functions. We, therefore, further examined the effects of these amino acid substitutions on E7 subcellular localisation to provide hints on possible underlying mechanisms of its oncogenic action. As HPV58‐containing cell line is unavailable, we expressed HPV58 E7 prototype and variants in human osteosarcoma U2‐OS cells, due to their HPV‐null background and high transfection efficiency. Confocal microscopy showed that HPV58 E7 prototype predominantly localized in the nucleus with a punctate pattern and could also be found in the cytoplasm, whereas HPV16 E7 distributed evenly throughout the cell in both cytoplasm^33^ and nucleus.^34^ Subsequent subcellular localisation studies revealed that all the three other HPV58 E7 variants, including V1 (T20I/G63S), displayed similarly nuclear punctates as prototype (Figure 4). We then undertook further immunofluorescence analyses to examine the identity of these nuclear punctates and found that they co‐localized with neither PML bodies nor γH2AX (data not shown). Therefore, our results indicated that all these variants retain their intracellular localisation and might have similar nuclear functions as prototype. Further investigation is deserved to understand the nature of the observed nuclear punctates.

#### 3.4.2 HPV58 E7 prototype and variants possessed a similar half‐life

**Figure 5:**
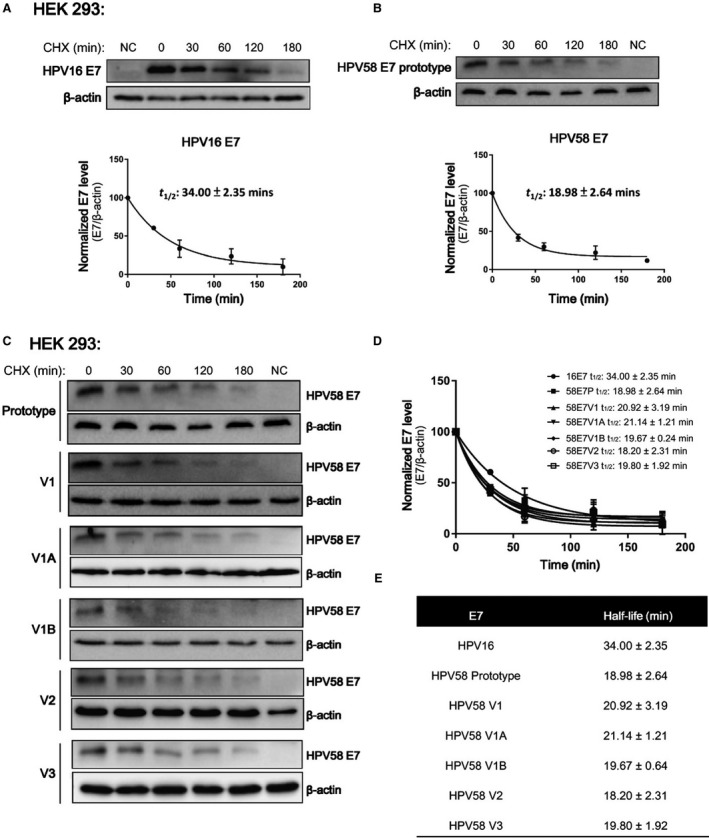
HPV58 E7 prototype and variants possess a similar half‐life, but shorter than of HPV16 E7. The effects of different variations on HPV58 E7 half‐life was examined by transfection of HPV58 E7 prototype (P), variants (V1: T20I/G63S; V2: G41R/G63D; V3: T74A/D76E) or artificial mutants (V1A: T20I only; V1B: G63S only) into HEK‐293 cells. After 24 h, 20 μg/mL cyclohexmide (CHX) was supplemented to halt protein translation and cell were then harvested at 0, 30 min, 1, 2 and 3 h. Actin served as loading control of the blots. The band intensities were quantitated by ImageLab and normalized with the corresponding actin level. The half‐life of E7 proteins was then calculated with the one phase exponential decay function using GraphPad™ Prism 7. (A) Consistent with published data, HPV16 E7 showed a half‐life of 34.0 ± 2.4 min. (B) HPV58 E7 had a significantly shorter half‐life of 19.0 ± 2.6 min. The upper panel shows a representative immunoblot with corresponding CHX treatment time indicated. The lower panel showed the one phase exponential decay curve of E7 plotted with data obtained from three independent experiments. (C) The half‐life of HPV58, regardless of the variations it carried, was approximately 19 min (ranging from 18.20 ± 2.31 to 21.14 ± 1.21 min). E7 half‐lives were calculated by using data from three independent experiments and expressed as mean ± SD minutes. (D) The one phase exponential decay curve of E7 and (E) the corresponding half‐life

We then further analysed whether amino acid variations of E7 may affect their protein stability using HEK‐293 cells by Western blotting. Consistent with published data that HPV16 E7 has a relatively short half‐life of about an hour,^33^ HPV16 E7 showed a half‐life of 34.0 ± 2.4 minutes in our study. Prototype HPV58 E7 had a significantly shorter half‐life of 19.0 ± 2.6 minutes (Figure 5A,B), and all the three HPV58 E7 variants, including V1 (T20I/G63S), had similar half‐lives to the prototype of approximately 19 minutes (ranging from 18.20 ± 2.31 to 21.14 ± 1.21 minutes). This implies that the studied amino acid variations do not affect E7 protein turn over (Figure 5C‐E).

### 3.5 HPV58 E7 V1 (T20I/G63S) degraded pRb more promptly, alike HPV58 E7 prototype

**Figure 6:**
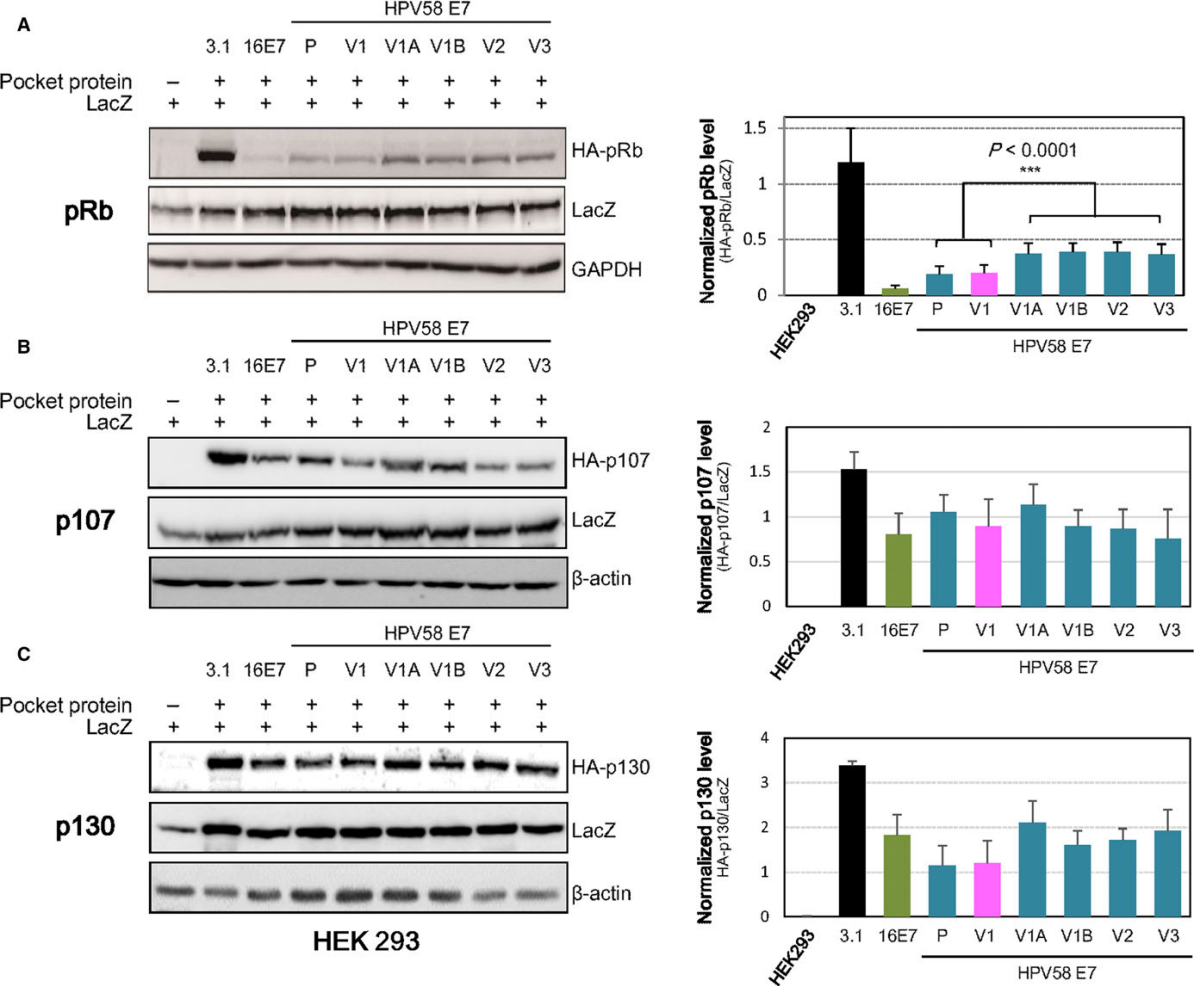
HPV58 E7 T20I/G63S variant exhibited stronger pRb degradation ability. The effects of HPV58 E7 variations on (A) pRb, and its related pocket protein (B) p107 and (C) p130 degradation were examined by cotransfection of HPV58 E7 prototype (P), variants (V1: T20I/G63S; V2: G41R/G63D; V3: T74A/D76E) or artificial mutants (V1A: T20I only; V1B: G63S only) together with pocket protein plasmid into HEK‐293 cells. LacZ was simultaneously cotransfected to normalize transfection efficiency among different groups. Results showed that HPV58 E7 (including prototype and all tested variants) could significantly degrade (A) pRb, (B) p107 and (C) p130 in HEK‐293 cells compared with cells transfected with vector and LacZ alone. (A) HPV58 E7 V1 variant and prototype exhibited significantly stronger pRb degradation power than other variants after normalising with the corresponding LacZ levels. HPV16 E7, which is known to readily degrade the pocket protein, served as a positive control of this assay. GAPDH or actin served as the loading control of the immunoblot. (B, C) There is no significant difference on p107 and p130 degradation between prototype and different variants, albeit a stronger degradation power of HPV58 E7 V1 and prototype on p130 was observed. The right panel showed a representative immunoblot image. Histogram on the left showed corresponding quantitation of band intensity normalized with LacZ using densitometry. Data are expressed as average ± SEM from three independent experiments. ***P <* *0.001

We then proceeded to characterize the possible underlying molecular mechanism contributing to the higher transforming potential of HPV58 E7 V1 (T20I/G63S) and prototype as demonstrated in the soft agar assay. Of the many cellular regulatory pathways that HPV16 E7 can subvert, the best characterized is targeting of pRb for degradation in a ubiquitin/proteasome‐dependent manner, which eventually stimulates cell cycle progression.^35^ It has been demonstrated that prototype HPV58 E7 can efficiently induce pRb degradation.^23^ To assess the effects of HPV58 E7 variations on degradation of pRb and related pocket protein (p107 and p130), the proteins were expressed exogenously in HEK‐293 cells and assessed by Western blotting. The HPV58 E7 V1 (T20I/G63S) and prototype induced markedly greater degradation of exogenous pRb than other variants by 26 ± 3% and 25 ± 3% respectively (*P* < 0.001, Figure 6A). Consistently, we observed that V1A (T20I) and V1B (G63S) can degrade pRb at a similar level as V2 (G43R/G63D) and V3 (T74A/T76E), even though at a weaker extent compared to V1 (T20I/G63S). This again indicated that both T20I and G63S were important for pRb recognition and hence, could contribute to higher oncogenic properties of V1.

On the other hand, though HPV58 E7 can degrade p107 and p130, there was no significant difference in p107 (Figure 6B) and p130 (Figure 6C) degradation between HPV58 E7 prototype and the different variants. However, it is perhaps worth noticing that both HPV58 E7 prototype and V1 (T20I/G63S) can degrade p130 at a better extent compared to HPV16 E7 and other HPV58 E7 variants in this experimental setting. Collectively, our results implied that HPV58 E7 prototype and V1 (T20I/G63S) preferentially degraded pRb over other pocket proteins; and did not confer additional oncogenicity through p107/p130 targeting.

## 4 DISCUSSION

While HPV16 and 18 remain to be the most important HPV types contributing to majority of cervical cancer cases globally, HPV58 has a unique oncogenic role in East Asia. As the oncogenic roles of HPV16 and 18 have been well‐established, this study aimed to focus on HPV58. In this connection, we have previously described the identification of several HPV58 E7 variants from clinical specimen; and among them, T20I/G63S (V1) was significantly associated with a higher risk for cervical cancer.^25^, ^27^ Herein, we sought to substantiate our earlier epidemiological observations by determining the biological significance of the HPV58 E7 variants, particularly V1 (T20I/G63S). We showed that while all tested E7 proteins demonstrated similar intracellular localisation and half‐life, the V1 (T20I/G63S) variant showed an increased immortalising and transforming ability with a higher pRb‐degrading power than the other two common circulating HPV58 E7 variants (V2 and V3).

Previous studies on HPV variants have focused on the most prevalent type, HPV16. In this regard, numerous studies have demonstrated that the epidemiologically high‐risk HPV16 E6 Asian‐American (AA) variant conferred a higher carcinogenic potential and, unlike other variants, its overexpression is sufficient to immortalize and transform primary human keratinocytes in the absence of E7.^36^ In contrast to HPV16, studies on HPV58 biology are scarce.^22^, ^23^ The only study on HPV58 variant available was performed to compare the transcriptional activity of the long control region of three HPV58 variants, and found that substitution of guanine to adenine residue at nucleotide position 7788 enhanced E6/E7 bistronic transcript promoter activity.^37^ Our current study represents the very first investigation to describe and compare the oncogenic properties of the three most common naturally occurring HPV58 E7 variants.

Our findings suggested that among the three most common circulating HPV58 E7 variants, the V1 (T20I/G63S) variant had a stronger immortalising/transforming ability and exerted higher oncogenicity through the pRb pathway. This is in concordance with our previous epidemiological findings that V1 possessed a stronger cancer risk association than V2 and V3.^27^ Structurally, the amino acid residue T20 resides in CR2, adjacent to the LXCXE domain, which is responsible for pRb binding (Figure 1A). It is believed that the major oncogenic function of E7 is mediated through degradation of the tumour suppressor pRb and its related pocket proteins p107 and p130.^35^ On the other hand, G63 lies within CR3, next to the CXXC domain of the zinc finger (Figure 1A). Thus, both amino acid residues locate very close to the two main functional domains of E7. From the biochemical point of view, substitution of threonine to isoleucine at amino acid residue 20 changes a polar residue to a non‐polar one, while substitution of glycine to serine at residue 63 does the reverse. It is anticipated that these two amino acid variations might change the overall isoelectric point and hydrophobicity of the protein, thus affecting the protein functions.

Although naturally occurring T20I and G63S variations always co‐exist,^27^ we generated artificial mutants in order to delineate their functional roles independent of each other. Our results showed that both T20I and G63S appeared to contribute to the observed oncogenic phenotypes and their combined effects were statistically stronger than that of individual variants. Interestingly, it is worth noting that the HPV58 E7 prototype, which is the very first HPV58 clone obtained from a Japanese woman suffering from invasive cervical carcinoma^28^ also showed a comparable colony‐forming ability in soft agar colony formation assay and possessed similar pRb‐degrading ability as the V1 (T20I/G63S) variant, indicating that they both possess higher oncogenic potential than the other studied HPV58 E7 variants (V2 and V3).

Interestingly, our previous epidemiological results found that the HPV58 E7 V1 (T20I/G63S) variant was more frequently detected in East Asia (34%) than other areas of the world (4.9%).^27^ Taken together with our current results which demonstrated a potential higher oncogenic role of the HPV58 E7 V1 variant, our study implicates the more common circulation of an oncogenic HPV58 E7 variant among the Asian population, which might partly explain the higher disease burden associated with HPV58 in East Asia.

In this study, we recruited cells from diverse genetic background (including murine primary cells and human cancer cell lines) to demonstrate the oncogenic potential of HPV58 E7. However, this study should be elucidated using physiological relevant human kertinocytes in future to confirm our results. In addition, ectopic expression has been used in our study to characterize the functional roles of HPV58 E7. Further investigations by stable expression to ensure a physiological dose of E7 to its binding partner is more favourable. Moreover, we have focused on E7 variations in this study, as our previous studies showed that sequence variations of HPV58 mainly reside in E7, while E6 is relatively conserved among individuals.^27^ Given that both E6 and E7 are major HPV oncoproteins, it is anticipated that co‐expression of E6 prototype with the E7 variants could give a more complete picture of HPV oncogenesis and might reveal more additional oncogenic properties that contribute to the increased cervical cancer risk of the E7 V1 (T20I/G63S) variant.

In summary, our studies successfully identified the HPV58 E7 V1 (T20I/G63S) variant and prototype as possessing higher carcinogenic potential than other variants in our experimental settings. As HPV58 accounts for 20% of CINI cases, more intensive cancer surveillance is recommended for carriers of the E7 V1 (T20I/G63S) variant and prototype, for early detection of the disease. The higher oncogenic potential of the E7 V1 (T20I/G63S) variant and prototype suggests that their carriers may present with a more malignant phenotype and a possible poorer disease outcome.

## AUTHORS’ CONTRIBUTIONS

PKSC conceived and designed the research study. PTYL, CH, GPYC, SSB and WCSH carried out experiments and collected the data. PTYL, SSB, CH, RWML, ZC, PM, MT and LB contributed to data analysis and interpretation. PTYL, CH and SSB generated the figures. PTYL wrote the manuscript, SSB and PKSC edited the manuscript. All the authors were involved in revising it and had final approval of the submitted version.

## CONFLICTS OF INTEREST

The authors confirm that there are no conflicts of interest.
